# Notes on leaf micromorphology of the rare herbaceous bamboo *Buergersiochloa bambusoides* Pilg. (Olyreae, Poaceae) from New Guinea and its taxonomic implications

**DOI:** 10.3897/phytokeys.172.59506

**Published:** 2021-02-19

**Authors:** Jamile F. Lima, Kelly Regina B. Leite, Lynn G. Clark, Reyjane P. Oliveira

**Affiliations:** 1 Departamento de Ciências Biológicas, Universidade Estadual de Feira de Santana, Avenida Transnordestina s/n, Novo Horizonte, 44036-900, Feira de Santana, BA, Brazil Universidade Estadual de Feira de Santana Feira de Santana Brazil; 2 Instituto de Biologia, Universidade Federal da Bahia, Rua Barão de Jeremoabo, 668, Campus de Ondina, 40170-115, Salvador, BA, Brazil Universidade Federal da Bahia Salvador Brazil; 3 Department of Ecology, Evolution, and Organismal Biology, Iowa State University, 251 Bessey Hall, Ames IA 50011-4009, USA Iowa State University Ames United States of America

**Keywords:** Branched papillae, bicellular microhair, Buergersiochloinae, saddle-shaped, silica body

## Abstract

We present notes on the leaf micromorphology of *Buergersiochloa bambusoides*, a rare species from New Guinea and included in Buergersiochloinae, one of three subtribes of the herbaceous bamboos (tribe Olyreae). We used scanning electron microscopy and light microscopy to analyze the microcharacters of both adaxial and abaxial leaf surfaces. Within the Olyreae, saddle-shaped silica bodies in both the costal and intercostal zones are considered unique to Buergersiochloinae. Simple, circular and very small papillae are observed on the adaxial surface, and for the first time, branched papillae on the abaxial surface are observed in *B. bambusoides*. On the abaxial surface, there are papillae on long cells associated with the stomatal complexes. Bicellular microhairs are the only trichomes present and they are found almost exclusively on the abaxial surface. The saddle-shaped silica bodies are the most taxonomically important among the microcharacters observed on the leaf surface of *B. bambusoides*.

## Introduction

*Buergersiochloa bambusoides* Pilg. is the only species of this genus historically included in Buergersiochloinae (Olyreae, Bambusoideae), which is endemic to the northern coastal rainforests of the Indonesian island of New Guinea (Fijten 1975) and Papua New Guinea (Judziewicz and Clark 2007; BPG 2012). This subtribe is sister to the clade composed of the other two lineages (Olyrinae and Parianinae), which are essentially restricted to the Neotropics (Oliveira et al. 2014; Ferreira et al. 2019). However, the recircumscription of Buergersiochloinae is in progress based on current phylogenetic studies (Carvalho et al. in press).

This is a rare, poorly collected and monoecious perennial species, with scaly branched rhizomes, leafy sterile culms and leafless fertile culms, and a rather dense paniculate synflorescence with male spikelets borne on the lower and female spikelets on the upper branches (Fijten 1975). Interestingly, although unisexual spikelets, cruciform silica bodies in the costal zone, and crenate silica bodies (olyroid-type) in the intercostal zone have been considered synapomorphies of the Olyreae, these types of silica bodies are absent in *B. bambusoides* (BPG 2012). A few previous observations on the leaf anatomy and micromorphology of this species were published by Renvoize (1985), in comparison to some genera of Olyrinae, using light microscopy (LM). However, there are no studies of this species using scanning electron microscopy (SEM), which clearly offers important information for understanding the foliar micromorphology in Olyreae (e.g. Oliveira et al. 2008a, b; Ferreira et al. 2013; Leandro et al. 2016; Lima et al. 2020), especially when compared to LM.

Papillae and trichomes can also inform general bamboo systematics (Calderón and Soderstrom 1967; Soderstrom and Ellis 1987; Leandro et al. 2020) and other microcharacters have been used in taxonomic studies within Olyreae (Metcalfe 1960; Calderón and Soderstrom 1967, 1973; Renvoize 1985; Jesus et al. 2012; Ferreira et al. 2013; Leandro et al. 2016; Lima et al. 2020), which remain undescribed for this species.

For these reasons, we present updated information on the foliar surfaces of this species, which was historically the single representative of the Buergersiochloinae. We also aim to answer the following specific questions regarding the leaf micromorphology of *B. bambusoides*: Do the silica bodies of the costal zone have the same orientation, with respect to the longitudinal axis of the leaf, like those of the intercostal zone? What is the morphology of the papillae and how are they distributed? On which cell type(s) are the papillae associated with the stomata found? What types of trichome occur? Do these characters have potential taxonomic utility?

## Materials and methods

Samples used in this work were obtained from the U.S. National Herbarium of the Smithsonian Institution (US) from the vouchers *Croft et al. 68692* and *Dransfield et al. 1382* [acronym according to Thiers (2020+)]. The epidermal micromorphology of the leaves of *B. bambusoides* was analyzed using scanning electron microscopy (SEM) and light microscopy (LM).

The SEM analysis was performed with two samples of 0.5 cm^2^ from the median region of fully developed dried leaf blades, to observe both adaxial and abaxial surfaces. Samples were submerged in xylene for ca. 10 minutes, eliminating the epicuticular wax to allow better observation of the microcharacters (Dávila and Clark 1990), and mounted on small steel cylinders with metallic adhesive tape and covered with platinum in a Denton Desk II sputter coater. They were imaged using a JEOL JSM-5800LV scanning electron microscope at the Roy J. Carver High Resolution Microscopy Facility at Iowa State University.

For the LM analysis, two other similar samples of the same size and from the same region of the leaf blades were used. The epidermises were dissociated using the Jeffrey method (Johansen 1940) and stained with 1% alcoholic safranin. Semipermanent slides were mounted with glycerinated gelatin, analyzed, and photomicrographed in a Zeiss Axio Scope A1 optical microscope with a Canon EOS attached digital camera. The description of the epidermal microcharacters followed the terminology proposed by Ellis (1979).

## Results

**Figure 1.**
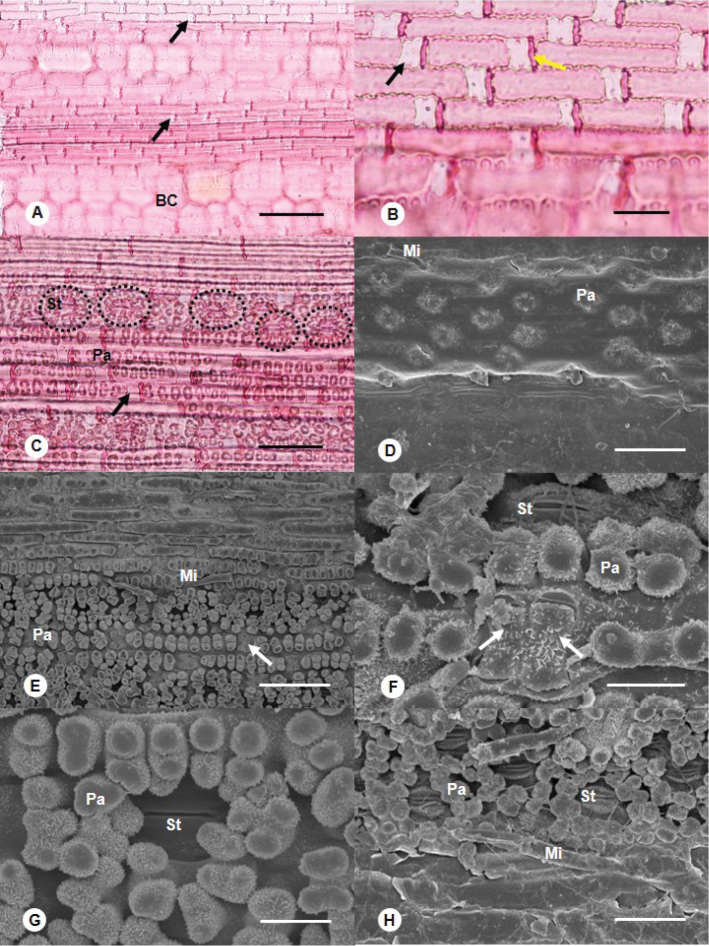
Leaf micromorphology of *Buergersiochloa bambusoides***A–C** leaf surface observed under LM**A** adaxial surface showing long cells, bulliform cells with sinuous anticlinal walls and saddle-shaped silica bodies **B** detail of saddle-shaped silica bodies and cork cells **C** abaxial surface showing silica bodies, abundant papillae and papillae encircling the stomata (dotted circles) **D–H** leaf surface observed under SEM**D** adaxial surface with very small papillae and the basal cell of a broken bicellular microhair **E** abaxial surface showing microhairs, abundant papillae on costal and stomatal cell rows, and long cell papillae encircling the stomata **F** detail of two adjacent saddle-shaped silica bodies, papillae and stoma on the abaxial surface **G** detail of papillae encircling a stoma **H** detail of panicoid type bicellular microhairs. BC: Bulliform cell; Mi: bicellular microhair; Pa: papilla; St: stoma. Black or white arrows: silica body; yellow arrow: cork cell. Scale bars: 100 µm (**A, C**); 25 µm (**B, H**); 10 µm (**D, G**); 50 µm (**E, F**)

**Epidermal cells**: long and tabular in the intercostal zones, and nearly equidimensional in the costal zones, walls anticlinally sinuous (Fig. 1A, B). **Short cells**: silica cells containing a silica body and cork cells with wall impregnated with suberin, the latter paired with silica cells (Fig. 1B) or the basal cells of bicellular microhairs. **Bulliform cells**: anticlinally sinuous wall, wider and shorter than long cells, in bands in the intercostal zone of the adaxial epidermis (Fig. 1A). **Stomatal distribution**: hypostomatic (Fig. 1C). **Silica bodies**: saddle-shaped and transversely elongated in the intercostal and costal zones (Fig. 1B). **Adaxial surface**: very small papillae on the bulliform cells; bicellular microhairs scarce, prickles, and macrohairs absent (Fig. 1D). **Abaxial surface**: stomata in 2 rows on each side of a costal zone; subsidiary cells dome-shaped, papillae absent; long cell papillae encircling the stomata in the stomatal cell rows (Fig. 1F); long cells bearing small, branched, abundant papillae in the costal and interstomatal cell rows; panicoid type bicellular microhairs present along the edge of stomatal cell rows; prickles and macrohairs absent (Fig. 1E, G, H).

## Discussion

The leaf blade epidermis of *B. bambusoides* is consistent with other members of Olyreae and indeed the whole subfamily, considering both long and short cells, in addition to the rows of bulliform cells in the adaxial intercostal zones (Vieira et al. 2002; Oliveira et al. 2008a, b; Jesus Junior et al. 2012; Leandro et al. 2016, 2020; Lima et al. 2020). The short cells observed include silica or cork ones, paired with each other or the latter paired with the basal cells of trichomes, as is common to Poaceae (Calderón and Soderstrom 1973; Soderstrom and Ellis 1988; Vieira et al. 2002; Lima et al. 2020).

The saddle-shaped silica bodies of *B. bambusoides* have been recognized as characteristic of many woody bamboos of the Arundinarieae and Bambuseae tribes (Renvoize 1987; Yang et al. 2008; Zhang et al. 2014; Leandro et al. 2020), but within the Olyreae are exclusive to Buergersiochloinae, as noted here and confirming previous studies (Renvoize 1985; Lima et al. 2020). For this reason, the presence of saddle-shaped silica bodies is here considered as an important taxonomic character for this subtribe since it does not have crenate silica bodies (olyroid-type) in the intercostal zone, which have been considered synapomorphies of the Olyreae (BPG 2012). In addition to the shape, the orientation of the silica bodies on the leaf surface is also considered important information for the identification and taxonomic utility of these microcharacters (Rudall et al. 2014). In *B. bambusoides* the silica bodies are transversely elongated (i.e. perpendicular to the long axis of the leaf), and have the same orientation in both the costal and intercostal zones. Thus, we confirm that they are the same type in both zones.

The simple and very small papillae on the adaxial leaf surface, found only on the outer periclinal wall of the bulliform cells in *B. bambusoides* may be related to the environment in which the plants occur (Lee 1986; Sahuquillo and Lumaret 1995; Oliveira et al. 2008a, b; Glover and Whitney 2010; Thomas et al. 2010; Ferreira et al. 2013). This species is endemic to lowland primary forests of New Guinea (Fijten 1975) and considering the shaded environment of the understory, these papillae can help to enhance the active capture of light (Lee 1986; Glover and Whitney 2010; Thomas et al. 2010) as suggested for *Eremitis afimbriata* F.M.Ferreira & R.P.Oliveira and *E. magnifica* F.M.Ferreira & R.P.Oliveira, the first registrations of this characteristic in bamboo leaves (Ferreira et al. 2013).

The stomatal complexes with dome-shaped subsidiary cells of *B. bambusoides* are very common in Bambusoideae (Metcalfe 1960; Ellis 1979; Zuloaga et al. 1993; Oliveira et al. 2008a, b; Jesus et al. 2012; Ferreira et al. 2013; Leandro et al. 2016, 2020; Lima et al. 2020), occurring in well-defined rows in the intercostal zones on either side of a costal zone. Although papillae on the subsidiary cells have been documented for some Olyreae, mainly members of subtribe Olyrinae (Lima et al 2020), and are also characteristic of Chusqueinae (Clark 1986; Soderstrom and Ellis 1987; Fisher et al. 2009, 2014), these are here indicated as absent in *Buergersiochloa*. The papillae associated closely with the stomatal complexes in this species are from the long cells of the stomatal rows, as is common in many woody bamboos (Yang et al. 2008; Guerreiro et al. 2013). However, these papillae on the long cells are branched and since they are observed for the first time on the abaxial surface of the leaf blade of *B. bambusoides*, this is a micromorphological novelty, not only for Buergersiochloinae, but also for Olyreae.

Bicellular microhairs observed on the abaxial surface were also similar to those often found in Bambusoideae as a whole (Prat 1936; Metcalfe 1960; Calderón and Soderstrom 1973; Ellis 1979; Vieira et al. 2002; Oliveira et al. 2008a, b; Jesus et al. 2012; Leandro et al. 2016; Lima et al. 2020), therefore classified as panicoid, having two cells that are longer than thick (Amarasinghe and Watson 1988), and the distal cell has a cellulosic wall that is sometimes lost (Tateoka et al. 1959).

## Conclusions

Among the microcharacters observed on the leaf surface of *B. bambusoides*, saddle-shaped silica bodies are the most taxonomically important, since this type of silica body was not observed in any genus of Olyrinae or Parianinae within Olyreae (Lima et al. 2020; Lima et al. unpubl. data).

The leaf surface of *B. bambusoides* presented simple and very small papillae on the adaxial surface, and branched papillae on the abaxial surface. This first record of branched papillae in *B. bambusoides* is a novelty for Olyreae. On the adaxial surface, the papillae are only on bulliform cells, and on the abaxial surface, the papillae of the long cells adjacent to stomata encircle the stomatal complexes. In *B. bambusoides* microhairs on the adaxial surface are very scarce but are more common on the abaxial surface, but because it is a type of trichome common in Bambusoideae, this character does not offer relevant taxonomic information.
